# Internal construct validity of the Warwick-Edinburgh Mental Well-being Scale (WEMWBS): a Rasch analysis using data from the Scottish Health Education Population Survey

**DOI:** 10.1186/1477-7525-7-15

**Published:** 2009-02-19

**Authors:** Sarah Stewart-Brown, Alan Tennant, Ruth Tennant, Stephen Platt, Jane Parkinson, Scott Weich

**Affiliations:** 1 Warwick Medical School, University of Warwick, Coventry, CV4 7AL, UK; 2 Department of Rehabilitation Medicine, Faculty of Medicine and Health, The University of Leeds, Leeds General Infirmary, St George St, Leeds, LS1 3EX, UK; 3 Coventry Teaching Primary Care Trust, Christchurch House, Greyfriars Lane, Coventry, CV1 2GQ, UK; 4 Community Health Sciences, School of Clinical Sciences & Community Health, University of Edinburgh, Teviot Place, Edinburgh, EH8 9AG, UK; 5 NHS Health Scotland, Elphinstone House, 65 West Regent Street, Glasgow, G2 2AF, UK

## Abstract

**Background:**

The Warwick-Edinburgh Mental Well-Being Scale (WEMWBS) was developed to meet demand for instruments to measure mental well-being. It comprises 14 positively phrased Likert-style items and fulfils classic criteria for scale development. We report here the internal construct validity of WEMWBS from the perspective of the Rasch measurement model.

**Methods:**

The model was applied to data collected from 779 respondents in Wave 12 (Autumn 2006) of the Scottish Health Education Population Survey. Respondents were aged 16–74 (average 41.9) yrs.

**Results:**

Initial fit to model expectations was poor. The items 'I've been feeling good about myself', 'I've been interested in new things' and 'I've been feeling cheerful' all showed significant misfit to model expectations, and were deleted. This led to a marginal improvement in fit to the model. After further analysis, more items were deleted and a strict unidimensional seven item scale (the Short Warwick Edinburgh Mental Well-Being Scale (SWEMWBS)) was resolved. Many items deleted because of misfit with model expectations showed considerable bias for gender. Two retained items also demonstrated bias for gender but, at the scale level, cancelled out. One further retained item 'I've been feeling optimistic about the future' showed bias for age. The correlation between the 14 item and 7 item versions was 0.954.

Given fit to the Rasch model, and strict unidimensionality, SWEMWBS provides an interval scale estimate of mental well-being.

**Conclusion:**

A short 7 item version of WEMWBS was found to satisfy the strict unidimensionality expectations of the Rasch model, and be largely free of bias. This scale, SWEMWBS, provides a raw score-interval scale transformation for use in parametric procedures. In terms of face validity, SWEMWBS presents a more restricted view of mental well-being than the 14 item WEMWBS, with most items representing aspects of psychological and eudemonic well-being, and few covering hedonic well-being or affect. However, robust measurement properties combined with brevity make SWEMWBS preferable to WEMWBS at present for monitoring mental well-being in populations. Where face validity is an issue there remain arguments for continuing to collect data on the full 14 item WEMWBS.

## Introduction

There is increasing international interest in the concept of positive mental health and its contribution to all aspects of human life [1,2]. The term is often used, in both policy and academic literature, interchangeably with the term mental well-being. It is a complex construct, which is generally accepted as covering both affect and psychological functioning as well as the overlapping concepts of hedonic and eudemonic well-being [3]. Positive mental health is recognised as having major consequences for health and social outcomes [4,5], and has given rise to new therapies that explicitly focus on facilitating positive mental health [6] and to health promotion programmes which aim to develop mental well-being at community level. The field of positive mental health is under-researched partly because of the lack of appropriate measures [7] and there is demand for instruments suitable for use with both individuals and populations.

The Warwick-Edinburgh Mental Well-Being Scale (WEMWBS) was developed to meet this demand [8]. It is an ordinal scale comprising 14 positively phrased Likert- style items. Development was undertaken by an expert panel drawing on the current academic literature, qualitative research with focus groups, and psychometric testing of an existing scale (the Affectometer 2). The new scale was validated on student and representative population samples in the UK using qualitative as well as quantitative methods and performed well against classic criteria for scale development [9]. WEMWBS showed good content validity, moderately high correlations with other mental health scales and lower correlations with scales measuring overall health. Its distribution was near normal and did not show ceiling effects in population samples. It discriminated between population groups in a way that is largely consistent with the results of other population surveys. Test-retest reliability at one week was high (0.83). Social desirability bias was lower than or similar to that of other comparable scales.

WEMWBS' internal scaling properties were tested using internal construct validity in the form of confirmatory factor analysis. Results were consistent with a single underlying construct. Internal consistency reliability was assessed using Cronbach's Alpha [10], which suggested item redundancy. In the context of testing scales based on ordinal data, it has been argued that both the latter approaches are inappropriate, given that factor analysis is parametric and requires interval scaling, and Cronbach's Alpha does not address unidimensionality [11-13]

Recently, modern psychometric approaches have been adopted to provide a more robust interpretation of the internal construct validity of ordinal scales, the most widely applied of which is the Rasch Measurement Model [14]. In this approach, data which include items intended to be summated into an overall ordinal score for a specific scale are tested against the expectations of this measurement model. These expectations are a probabilistic form of Guttman Scaling which operationalises the formal axioms that underpin measurement [15,16]. Other issues such as category ordering (do the categories of an item work as expected?) and item bias, or Differential Item Functioning (DIF) [17] may also be addressed within the framework of the Rasch model. Finally, when data are found to fit model expectations a linear transformation of the raw ordinal score is obtained, opening up valid parametric approaches, given appropriate distributions [18,19].

In this report we assess the internal construct validity of the 14-item Warwick-Edinburgh Mental Well-being Scale (WEMWBS) from the perspective of the Rasch Measurement Model using data collected from Wave 12 (Autumn 2006) of the Scottish Health Education Population Survey (HEPS).

## Methods

### The Warwick-Edinburgh Mental Well-being Scale (WEMWBS)

WEMWBS differs from other scales of mental health in that it covers only positive aspects of mental health and all 14 items are phrased positively (see additional file 1). Items cover a range of aspects of mental well-being including many which will be familiar from other well known scales (e.g. I've been feeling relaxed, I have been thinking clearly). Responses in the form of a Likert scale comprise 'None of the time'; 'Rarely'; 'Some of the time'; 'Often' and 'All of the time'. Scores range from 14 to 70, with a higher score reflecting a higher level of mental well-being.

### The Health Education Population Survey (HEPS)

HEPS was a Scottish population survey in which data were collected on an annual basis in two waves (Spring and Autumn) from a representative sample of the adult population aged 16 to 74. Conducted from 1996 to 2007, HEPS has subsequently been decommissioned and replaced by a module in the Scottish Health Survey 2008. NHS Health Scotland commissioned HEPS and fieldwork was carried out by BMRB International.

Data for this validation study came from Wave 12 (Autumn 2006) of the survey. Allowing for invalid addresses, a response rate of 66% was achieved. Interviews were carried out face to face, in people's homes, using Computer Assisted Personal Interviewing. In this data set 779 respondents completed all or part of WEMWBS, of whom 45.8% were male. The average age of respondents (767 with continuous age information) was 41.9 years (SD 16.05) and the range 16–74 years. As the Rasch analysis (see below) bases person estimates upon the information that is available, estimates can be given where missing values are present. However, the precision of the estimate is reduced to an extent depending on the number of missing items.

### The Rasch model

In satisfying the axioms of conjoint measurement [20], the Rasch model shows what is expected of responses to items in a scale if measurement (at the metric level) is to be achieved. Dichotomous [14] and polytomous versions of the model are available [21,22]. The model assumes that the probability of a given respondent affirming an item is a logistic function of the relative distance between the item location and the respondent location on a linear scale. In other words the probability that a person will affirm an item is a logistic function of the difference between the person's level of, for example, mental well-being, and the level of well-being expressed by the item. The model can be expressed in the form of a logit model:

$$\ln\left(\frac{P_{ni}}{1-P_{ni}}\right)=\theta_{n}-b_{i}$$

where ln is the normal log, P is the probability of person *n *affirming item *i; θ *is the person's level of mental well-being, and *b *is the level of mental well-being expressed by the item.

The process of Rasch analysis is described in detail elsewhere [23,24]. Briefly, the analysis is concerned with how far the observed data match that expected by the model, using a number of fit statistics. In this paper, three overall fit statistics are considered. Two are item-person interaction statistics transformed to approximate a z-score, representing a standardised normal distribution. Therefore if the items and persons fit the model, a mean of approximately zero and a standard deviation of 1 would be expected. A third is an item-trait interaction statistic reported as a Chi-Square, reflecting the property of invariance across the trait. A significant Chi-Square indicates that the hierarchical ordering of the items varies across the trait, so compromising the required property of invariance.

In addition to these overall summary fit statistics, individual person- and item-fit statistics are presented, both as residuals (a summation of individual person and item deviations) and as a Chi Square statistic. In the former case residuals between ± 2.5 are deemed to indicate adequate fit to the model. To take account of multiple testing Bonferroni corrections are applied to adjust the Chi-square p value [25]. The same fit statistics are available to detect person deviation, as a few respondents significantly deviating from model expectation may cause significant misfit at the item level.

The proper ordering of response categories is also evaluated. Failure to follow an expected increase in response option consistent with an underlying increase in mental well-being would show disordered thresholds across categories within an item. The term *threshold *refers to the point between two response categories where either response is equally probable. For a given item the number of thresholds is always one less than the number of response options.

Within the framework of Rasch measurement, the scale should also work in the same way irrespective of which group (e.g. gender) is being assessed [26]. For example, in the case of measuring mental well-being, males and females should have the same probability of affirming an item (in the dichotomous case), *at the same level of mental well-being*. Thus the probability is conditioned on the trait. If for some reason one gender did not display the same probability of affirming the item, then this item would be deemed to display differential item functioning (DIF), and runs the risk of biasing results. For example, if items were biased for gender, then gender could not be used as a predictor variable for mental well-being, as the measurement of mental well-being would be confounded by gender bias. It is important to note that the detection of and, if necessary, the adjustment for DIF, does not remove the effect of gender, but rather ensures that there is no gender bias in the scale so that the effect of gender can be properly understood. In practice adjustments for such bias can be made post-hoc in most circumstances, but items displaying DIF would be prime candidates for removal in any scale revision [27]. Sometimes bias may cancel out in the test, for example, one item may favour males, another females, and their effects may be nullified [28]. In the current analysis, DIF was tested for age, gender, and the presence or not of a long-standing illness.

Strict tests of unidimensionality are undertaken at every stage of analysis [29]. A Principal Component Analysis (PCA) of the residuals is undertaken, the standardised person-item differences between the observed data and what is expected by the model for every person's response to every item. After extracting the 'Rasch factor' there should be no further pattern in the data. This is formally tested by allowing the factor loadings on the first residual component to determine 'subsets' of items and then testing, by an independent *t*-test to see if the person estimate (the logit of person 'ability' or, in this case 'mental well-being') derived from these subsets significantly differ from each other [29,30]. If more than 5% of independent t-tests are found to be significant, allowing for a Binomial confidence interval for a proportion, this would indicate a breach of the assumption of unidimensionality.

An estimate of the internal consistency reliability of the scale is also available, based on the Person Separation Index (PSI) where the estimates on the logit scale for each person are used to calculate reliability. This is equivalent to Cronbach's Alpha [10].

In order to obtain robust estimates of the internal construct validity of the scale, the total data set is randomised into two further sets of approximately 50% of cases. Final results concerning the validity of the scale should be robust over the full data set, and each random sample.

The Rasch analysis was undertaken with the RUMM2020 software package [31].

## Results

**Figure 1:**
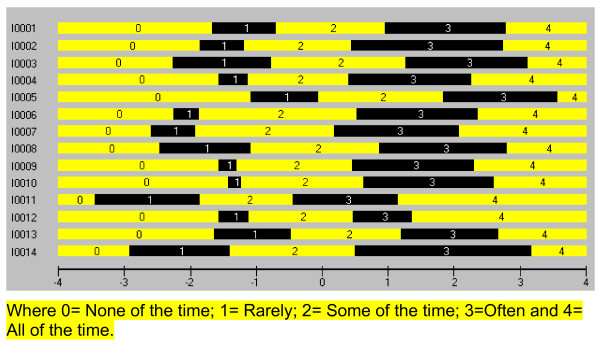
**Threshold map for the 14 item scale**. (See additional file 1 for full text of items).

The 779 cases initially displayed no floor or ceiling effects, and thus all were entered into the analysis. The log Likelihood test Chi Square was 143.75 (df 38) with a probability < 0.0001, indicating that the partial credit version of the Rasch model was appropriate. All thresholds were found to be ordered (Figure 1). That is, within each item, the transition from one category to the next represents an increase in the underlying trait of mental well-being.

Initial fit to model expectations was poor (Table 1 – Analysis 1). The items 'I've been feeling good about myself', 'I've been interested in new things' and 'I've been feeling cheerful' all showed significant misfit to model expectations, and were deleted. This led to a marginal improvement in fit (Analysis 2). A further two items 'I've been feeling interested in other people' and 'I've had energy to spare' were deleted, resulting in further improvement (Analysis 3).

**Table 1:** Fit of data to the Rasch model.

	Item		Person		Chi Square Interaction		PSI	Unidimensional *t*-tests (Confidence intervals)
Analysis^μ^	Mean	SD	Mean	SD	Value (df)	p		

1	0.102	3.111	-0.533	1.730	312.1 (126)	0.000	0.906	11.68% (10–13%)

2	0.102	2.084	-0.504	1.555	178.9 (99)	0.000	0.879	9.15% (8–11%)

3	0.143	1.580	-0.491	1.448	114.2 (85)	0.009	0.872	7.03% (6–9%)

4	0.080	1.794	-0.472	1.295	87.19 (63)	0.023	0.840	4.17%*

5	0.065	1.341	-0.475	1.222	64.70 (54)	0.151	0.845	4.18%*

6	0.126	0.681	-0.472	1.223	41.1 (54)	0.901	0.837	4.77%*

7	0.113	1.436	-0.437	1.194	56.5 (54)	0.382	0.854	5.15% (3–7%)

8	0.078	2.036	-0.540	1.743	208.7 (126)	0.000	0.903	11.77% (10–13%)

9	0.262	2.372	-0.472	1.656	233.3 (126)	0.000	0.910	10.67% (9–12%)

* Confidence intervals not relevant where values are <5%

^μ^Key to analysis

1 14 items

2 11 items

3 9 items

4 7 items

5 7 items DIF cancelled

6 Analysis 5 tested on sample 1

7 Analysis 5 tested on sample 2

8 14 items males

9 14 items females

Local dependency was then observed for two more items and, after further analysis, a strict unidimensional seven item scale was resolved (Analysis 4), comprising:

Item 1 – I've been feeling optimistic about the future

Item 2 – I've been feeling useful

Item 3 – I've been feeling relaxed

Item 6 – I've been dealing with problems well

Item 7 – I've been thinking clearly

Item 9 – I've been feeling close to other people

Item 11 – I've been able to make up my own mind about things

We have named this shortened scale SWEMWBS (Short Warwick-Edinburgh Mental Well-being Scale) (see additional file 2).

**Figure 2:**
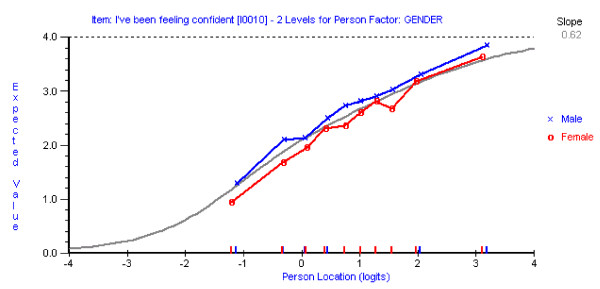
**Differential Item Functioning by Gender for the item 'I've been feeling confident'**.

Five out of the seven items discarded showed significant DIF for gender (Table 2). For example, the item 'I've been feeling confident' (item 10) showed that, at any level of mental well-being, males were more likely to report a higher score than females (Figure 2).

**Table 2:** Differential Item functioning for gender

Item	Main Effect for Gender				Gender-Trait Interaction			
	Males	Females	DF	Prob	Males	Females	DF	Prob
1	0.04015	0.04142	1	0.838742	1.09147	1.12607	9	0.341345
2	1.97595	2.14194	1	0.143738	1.00945	1.09425	9	0.364425
3	8.80378	9.87322	1	**0.001733**	0.30942	0.34701	9	0.958872
4	33.40524	30.25532	1	**0.000016**	0.91752	0.831	9	0.587624
5	13.15898	12.5703	1	**0.000431**	1.95187	1.86455	9	0.054061
6	1.64136	1.94816	1	0.163188	0.74287	0.88173	9	0.541148
7	3.22218	3.97158	1	0.046619	1.51676	1.86952	9	0.053306
8	8.52653	12.92955	1	**0.000355**	0.47544	0.72096	9	0.689971
9	20.25451	21.9388	1	**0.000019**	0.1508	0.16334	9	0.997325
10	20.48471	26.80281	1	**0.000000**	0.03225	0.0422	9	0.999991
11	0.3434	0.3741	1	0.540959	0.93481	1.01839	9	0.423315
12	16.02162	14.70792	1	**0.000147**	0.41747	0.38324	9	0.943387
13	1.4163	1.16839	1	0.280081	1.29212	1.06595	9	0.385782
14	0.39479	0.59179	1	0.441960	0.79332	1.18918	9	0.298521

Emboldened probabilities show significant DIF. Shaded items are those that were deleted.

Item numbers correspond to the order of items in WEMWBS (additional file 1)

In the final seven item scale two items also showed DIF for gender, but these were found to cancel out at the test level, and fit improved further (Analysis 5). One further item (item 1) 'I've been feeling optimistic about the future' still displayed marginal DIF for age. None of the items in the 14 item WEMWBS showed DIF by the presence or absence of a long-standing condition. As might be expected with a shorter scale, the level of reliability had fallen from 0.906 (Analysis 1) to 0.845 (Analysis 5), although the original 14 item version is compromised by multidimensionality caused by gender bias.

Given satisfactory fit to the Rasch model for the seven item scale, and confirmation of strict unidimensionality, the robustness of the solution (analysis 5) was tested on the two random samples embedded within the data (Analyses 6 & 7). Both subsets of data showed good fit to model expectations. A linear transformation of the raw score, based upon the seven valid items, was then made. The raw score-logit transformation is given in Table 3. The Spearman's correlation between the raw scores of WEMWBS and SWEMWBS was 0.954.

**Table 3:** Raw score to metric score conversion table for SWEMWBS.

Raw Score	Metric Score
7	7.00

8	9.51

9	11.25

10	12.40

11	13.33

12	14.08

13	14.75

14	15.32

15	15.84

16	16.36

17	16.88

18	17.43

19	17.98

20	18.59

21	19.25

22	19.98

23	20.73

24	21.54

25	22.35

26	23.21

27	24.11

28	25.03

29	26.02

30	27.03

31	28.13

32	29.31

33	30.70

34	32.55

35	35.00

Finally, given the disturbance in model fit brought about by bias associated with gender, the data from the full 14 item scale was fitted to the Rasch model independently for each gender. Neither the males (Analysis 8) nor the females (Analysis 9) demonstrated fit to model expectations, suggesting that the disturbance to the scale was more than just gender DIF.

## Discussion

Increasingly, scales used for measuring health and medical outcomes are being developed to meet the strict criteria associated with additive conjoint measurement as operationalised through the Rasch measurement model [14,20]. Providing a scientific basis for the construction of linear measurement this approach is now widely used in the health and social sciences [32,33]. It remains true, however, that the majority of scales commonly used to measure mental health in trials and population surveys have not been shown to meet these strict criteria.

Our analysis has shown that seven of the original 14 items of WEMWBS, which we have called SWEMWBS (Short Warwick-Edinburgh Mental Well-being Scale), conform to Rasch model expectations and provide a valid raw score – interval level transformation with a correlation of 0.954 to the full scale. Furthermore, SWEMWBS has been shown to be largely free of item bias, and that its polytomous response structure works as intended, with higher scores within an item reflecting greater overall mental well-being.

Although confirmatory factor analysis (not shown) had indicated that WEMWBS was consistent with a single underlying factor [8] the scale did not meet the criteria required of the Rasch model. Most of the seven items excluded showed bias for gender. Perhaps because of this DIF (which can be a cause of multidimensionality), it was not possible to construct a second meaningful scale from the seven deleted items. Separate analyses of the 14 item set by gender showed lack of fit to model expectations on both occasions, suggesting an underlying problem over and above the disturbance caused by gender DIF. In order to satisfy the rules for constructing interval scaling, the Rasch model imposes the strictest measurement criteria and. WEMWBS lack of fit to model expectations may have arisen either because of dimensionality issues, or because of the additional requirements for interval scale measurement over and above that required for ordinal scales.

WEMWBS was developed, in part, to support the evaluation of mental well-being programmes. The latter involve a component of education about the nature of mental well-being, which for many members of the public is a new concept. For this reason it was considered important that WEMWBS presented a full picture of mental well-being including items relating to the majority of aspects proposed in the academic literature. Face validity studies with the general public and its popularity with those practicing mental health promotion and public mental health in the UK suggest that WEMWBS met this goal.

In terms of face validity, the 7 item scale (SWEMWBS) presents a more restricted view of mental well-being than the 14 item scale (WEMWBS), with most items representing aspects of psychological and eudemonic well-being, and few covering hedonic well-being or affect. In terms of measurement properties, however, the 7 item scale (SWEMWBS) was robust to Rasch model expectations, whereas the original 14 item scale (WEMWBS) was not. The lack of measurement validity shown by half the items in the original 14 item scale may be attributable to current levels of knowledge and self-awareness relating to mental well-being among the general public resulting in responses which are not robust. As knowledge and self awareness increase this situation may change.

Given that SWEMWBS is embedded within the larger WEMWBS, it may be appropriate to continue to collect data on the full 14 items to further investigate dimensionality and gender bias in different samples. It would also allow for comparison, at the ordinal level, with earlier studies. However, our results clearly indicate that the 7 item scale is preferable to the 14 item scale where robust interval scale measurement is important, and respondent burden is an issue. To facilitate this, we have been able to provide a raw-score to interval scale transformation of the 7 item scale for use when change scores and other parametric procedures are required.

## Conclusion

Although providing a broader view of mental well-being than the shortened version (SWEMWBS), WEMWBS does not meet the strict criteria for measurement demanded by the RASCH model, demonstrating DIF and multidimensionality. The shortened scale, comprised of 7 items (SWEMWBS), satisfied all criteria, including strict unidimensionality. A linear transformation of the raw score from SWEMWBS (Table 3) can be used with confidence in parametric analyses, given appropriate distribution. Responses to mental well-being scales may change as knowledge and self-awareness increase at population level. There are, therefore, arguments for continuing to gather data on the 14 item scale (given the seven item scale is embedded) to examine measurement of mental well-being at the ordinal level, to explore item bias in different samples, and to further analyse potential dimensionality.

## Competing interests

This research was commissioned by NHS Health Scotland.

## Authors' contributions

SSB conceived of the study, supported the study design, coordinated the development of the instrument and drafted the manuscript. AT carried out all the statistical analyses and produced the first draft of the manuscript. RT designed and coordinated the study. SP participated in the design and coordination of the study, and helped to draft the manuscript. JP commissioned the study, participated in its coordination and helped to draft the manuscript. SW participated in the coordination of the study and helped to draft the manuscript.

## Supplementary Material

Additional file 1 **WEMWBS. **Warwick-Edinburgh Mental Well-being Scale. Click here for file

Additional file 2 **SEMWBS.** The Short Warwick-Edinburgh Mental Well-being Scale. Click here for file
