# Supplementary material for: Internal construct validity of the Warwick-Edinburgh Mental Well-being Scale (WEMWBS): a Rasch analysis using data from the Scottish Health Education Population Survey
Source: Health Qual Life Outcomes. 2009 Feb 19;7:15. doi: 10.1186/1477-7525-7-15 (PMC2669062; doi:10.1186/1477-7525-7-15)
Supplement: Additional file 2 — SEMWBS. The Short Warwick-Edinburgh Mental Well-being Scale. [file 1477-7525-7-15-S2.doc]

**The Short Warwick-Edinburgh**

**Mental Well-being Scale**

**(SWEMWBS)**

**Below are some statements about feelings and thoughts.**

**Please tick the box that best describes your experience of
each over the last 2 weeks**

| **STATEMENTS** | **None of the time** | **Rarely** | **Some of the time** | **Often** | **All of the time** |
| --- | --- | --- | --- | --- | --- |
| I’ve been feeling optimistic about the future | **1** | **2** | **3** | **4** | **5** |
| I’ve been feeling useful | **1** | **2** | **3** | **4** | **5** |
| I’ve been feeling relaxed | **1** | **2** | **3** | **4** | **5** |
| I’ve been dealing with problems well | **1** | **2** | **3** | **4** | **5** |
| I’ve been thinking clearly | **1** | **2** | **3** | **4** | **5** |
| I’ve been feeling close to other people | **1** | **2** | **3** | **4** | **5** |
| I’ve been able to make up my own mind about things | **1** | **2** | **3** | **4** | **5** |

“Short Warwick Edinburgh Mental Well-Being Scale (SWEMWBS)

© NHS Health Scotland, University of Warwick and University of Edinburgh, 2008, all rights reserved.”
